# Adherence to recommended physical activity restrictions due to threatened preterm delivery – a descriptive multi-center study

**DOI:** 10.1186/s12884-023-05371-5

**Published:** 2023-01-24

**Authors:** Jane M. Bendix, Mette G. Backhausen, Hanne K. Hegaard, Ane Lilleoere Rom, Stig Molsted, Ellen C. L. Lokkegaard

**Affiliations:** 1 Department of Gynecology and Obstetrics, Copenhagen University Hospital - North Zealand, Hillerod, Denmark; 2 Department of Clinical Research, Copenhagen University Hospital - North Zealand, Hillerod, Denmark; 3 Department of Gynecology and Obstetrics, Zealand University Hospital, Roskilde, Denmark; 4 Department of Obstetrics, Copenhagen University Hospital - Rigshospitalet, Copenhagen, Denmark; 5 Department of Clinical Medicine, University of Copenhagen, Copenhagen, Denmark; 6 The Interdisciplinary Research Unit of Women’s, Children’s and Families’ Health, The Juliane Marie Centre for Women, Children and Reproduction Copenhagen University Hospital - Rigshospitalet, Copenhagen, Denmark; 7 Research Unit of Gynaecology and Obstetrics, Department of Clinical Research, University of Southern Denmark, Odense, Denmark

**Keywords:** Threatened preterm delivery, Activity restrictions, Adherence, Accelerometric data, Physical positions/movements, Admission status

## Abstract

**Background:**

Threatened preterm delivery is a serious obstetrical complication and has for decades been prescribed physical activity restrictions (AR). Adherence to the recommended level of physical AR is however unknown. This study aimed to assess the objectively measured different physical positions and activities of pregnant women recommended AR due to threatened preterm delivery complications, compared to a reference group of uncomplicated pregnant women without restrictions, and to explore if admission status influenced adherence to AR.

**Methods:**

A Danish descriptive, clinical multi-center study included singleton pregnancies between 22–33 gestational weeks admitted to an antenatal ward or during midwife consultations either prescribed AR due to threatened preterm delivery or uncomplicated controls without restrictions. For seven days participants wore two tri-axial accelerometric SENS® monitors. Accelerometric data included time spent in five different positions, activities, and step counts. At inclusion demographic and obstetric information was collected.

**Results:**

Seventy-two pregnant women participated; 31% were prescribed strict AR, 15% moderate, 3% light, 8% unspecified, and 43% had no AR. Strict AR participants rested in the supine/lateral position for 17.7 median hours/day (range:9.6–24.0); sat upright 4.9 h/day (0.11–11.7); took 1,520steps/day (20–5,482), and 64% were inpatients. Moderate AR participants rested in the supine/lateral position for 15.1 h/day (11.5–21.6); sat upright 5.6 h/day (2.0–9.3); took 3,310steps/day (467–6,968), and 64% were outpatients. Participants with no AR rested 10.5 h/day (6.3–15.4) in supine/lateral position; sat upright 7.6 h/day (0.1–11.4) and took 9,235steps/day (3,225–20,818). Compared to no restrictions, participants with strict or moderate AR spent significant more time in physical resting positions and took significant fewer mean steps. Among strict AR admission status did not alter time spent in the physical positions, nor the step count.

**Conclusions:**

Overall, participants adhered highly to the recommended AR. However, discriminating between strict and moderate AR recommendations did not alter how physical resting positions and activities were carried out. The admission status did not influence how participants adhered to strict AR.

## Introduction

Preterm deliveries are the leading cause of mortality among children under the age of five years. Moreover, it is associated with more than half of long-term morbidity and causes large health-care costs [1, 2].

The prevalence of preterm delivery varies greatly worldwide; in many global regions the rate is more than 10% whereas in the Nordic and Baltic countries the rate is 5–7% [3]. These variations as well as the pathophysiology of preterm delivery are poorly understood [1, 3, 4].

In lack of an effective treatment to prevent or postpone preterm delivery, the clinical practice has been for decades to recommend physical activity restrictions/immobility/bed rest even though the practice has no evidence based documentation [5–7]. Lately a general counsel has been stated against routinely recommended AR as a treatment to reduce preterm birth [8, 9]. Nonetheless, there is still a tendency in the clinical practice to recommend AR [6, 7, 10]. In Denmark AR is yet recommended in threatened preterm deliveries prior to gestational week 28 or in case of preterm prelabor rupture of membranes (PPROM) with a cervix less than 25 mm [11].

Despite the lack of evidence even now when AR is recommended the definition ranges from a few hours of daily rest in a supine or laid back position during the waking hours of the day to rest around the clock except for meals and when having a bath and during toilet visits [6, 12]. Treatment with AR is often divided into light, moderate or strict AR. Light AR involves ≤ 2 h of rest; moderate AR > 2–8 h, while strict AR involves rest around the clock. If prescribed moderate or strict AR, neither household chores nor any kind of lifting are recommended [6, 12]. In general, pregnant women with threatened preterm delivery before gestational week 34 will be recommended strict AR in case of shortened cervix, with or without contractions, preterm prelabor rupture of membranes (PPROM) and/or vaginal bleeding. Moderate or light AR will often be recommended if e.g., the women have many or regular uterine contractions / Braxton Hicks contractions without shortening of the cervix, preeclampsia, or foetal growth restriction [6, 7].

However, neither the effect nor the objective level of AR has been documented [1, 12–14]. In contrast, it is evident that AR as an obstetric treatment regimen causes several significant physical and psychological side effects for the pregnant women involved as well as their families [1, 12–18]. When pregnant women are recommended AR, they are presumed to adhere to the recommended level of AR regardless of whether it takes place during hospitalization as inpatient or at home as outpatient. It is, however, unknown to what degree they adhere to the recommended level of AR and how AR impacts their daily physical positioning and activities.

Hence, the aim of this study was to assess the objectively measured different physical resting positions and activities of pregnant women recommended different levels of AR due to threatened preterm delivery complications, compared to a reference group of uncomplicated pregnant women without restrictions, and to explore if the admission status influenced adherence to AR.

## Material and methods

This clinical descriptive study was prospectively performed at three different tertiary Danish hospitals in the Capital and the Zealand regions between February 2019 and October 2020. Yearly the three hospitals take care of between 2500 and 6000 deliveries. In Denmark the rate of preterm deliveries before gestational age 34 is approximately 1.5% and a diagnosis of threatened preterm delivery explains 31% of all antenatal obstetric hospitalisations [19]. The use of recommended AR is not an intervention with a specific treatment or discharge code and its general prevalence is therefore unknown [1].

Women with singleton pregnancies, who were assigned to give birth at the included hospitals were eligible to participate in the study from gestational age of 22 to 33 if they had been recommended AR due to a diagnosis of threatened preterm delivery (preterm contractions with or without tocolysis treatment, short cervix < 20 mm assessed by cervical ultrasonic scan, vaginal bleeding, and/or PPROM) or if they had uncomplicated pregnancies and no recommended AR. Moreover, inclusion criteria allowed no allergies to band aid, no chronic diseases that affected the daily level of physical activities, and no immediate preterm delivery (no fibronectin testing were required according to Danish guidelines [20]). Further, participants had to be 18 years or older of age and be able to understand Danish in speaking and writing.

Recruitment took place at outpatient antenatal midwife clinics as well as at the emergency antenatal wards. The attending midwifes informed eligible women orally and in writing about the study. If a woman was interested a researcher approached her and informed in detail about the study. If accepting participation, the woman gave informed written consent and was then included in the study.

For seven days the study participant wore two accelerometric *SENS motion®* sensors attached with Micropore 3 M® band aid on the right side of the chest 3-4 cm below the clavicle and lateral from the sternum, and laterally on the right thigh 10 cm above the patella. The devices are waterproof, and the participants were therefore instructed not to remove them during bathing, swimming, and showering. During the study period participants could change the band aid if needed as additional Micropore 3 M® band aid and an instruction sheet was provided at the inclusion.

At inclusion the two accelerometric *SENS motion®* sensors were attached. If possible, the accelerometric data collection was initially validated by the registration of the five different physical positions and activities, each manually registered for 3 min by the researcher.

At inclusion demographic and obstetric data were collected including maternal age, parity, gestational age at inclusion, pregnancy complication, pregestational body mass index, educational level, work status, smoking status, partner status, and level of self-reported pregestational physical activity (high (competitive sports); moderate; light; sedentary) [21]. Moreover, the admission status i.e. inpatient, outpatient, or combined in-/outpatient AR was registered at inclusion and during the data collection period.

Recommended AR was classified as strict, moderate and light respectively as described in details elsewhere [6]. In brief, strict AR was in this study defined as supine or laid-back rest most of the waking hours of the day (> 8 h daily) except for meals and bathroom use; moderate AR as > 3-8 h rest and light AR as 3 h daily rest. It was the participant’s perception of the recommended restriction level that classified the AR, as the restriction level seldom was specified in the medical files. If the specific level (strict or moderate) of prescribed recommended AR was unclear to a participant, it was classified as ‘unspecified’.

### The accelerometric data from *SENS motion*® sensors

The *SENS motion®* sensors collected accelerometric data of time spent in supine or lateral rest, upright sitting, upright standing, sporadic gait, walking and the number of steps taken [22]. Hence, the study gathered data as a continuum of five different positions/movements between physical inactivity and physical activity. The measurements of time spent in the different physical positions and activities were recorded continuously throughout study participation by the two miniature tri-axial accelerometers (dimensions: 50 × 21 × 5 mm, weight: eight grams; SENS motion® PLUS activity measurement system, version 1.7.6). The system measures movements continuously at 12 Hz, 24 h a day. Data were analyzed in 5-s epochs, which each was estimated to belong to a certain physical activity category. The activity intensity was calculated as the average vector magnitude of the high-pass filtered 3 axis accelerometer measurements at 12 Hz sampling frequency subtracted the noise present on each measurement axis, done on each 5-s epoch.

### Statistical analyses

Prior to data analyses a qualitative audit was performed by two of the researchers (JMB, MGB) in one third of the accelerometric data sets, which were randomly selected. To assure exact accelerometric measurements a qualitative comparison of the validation data and the study data was done and showed excellent compliance with 100% agreement in all cases.

Demographic, obstetric and accelerometric data were stratified according to level of recommended AR and presented as number (%) or median value (range min–max). The individual accelerometric data were plotted in different scatterplots stratified by level of recommended AR.

Linear regression models were used to test differences between the different gradations of AR. In two steps a general linear regression analysis was performed with the independent variables strict AR versus no AR and with moderate AR versus no AR. Adjustment was made for the a priori defined factors known to clinically influence time spent in physical inactive positions and in physical activities during pregnancy including gestational age, parity, admission status, educational status, and time monitored. Mann Whitney was performed to test the difference between strict AR during inpatient admission status and strict AR during combined in-/outpatient admission status. These data are presented as β [95% CI].

The statistical significance was defined as a *p* value of less than 0.05. All analyses were performed using SPSS 25 software (IBM. Corp., Denmark, Europe).

### Ethical approval

The study was approved by The Danish Data Protection Agency (VD-2018–305, I-Suite 6591). The Danish Regional Committee on Biomedical Research Ethics was notified about this study and the committee decided that ethical approval was unnecessary (Notification Request no. 18021398). The respective administrations of the obstetric departments at the three study sites all approved the study. At inclusion all participants gave written informed consent to participate.

All methods were carried out in accordance with the study protocol and other relevant guidelines and regulations.

## Results

**Fig. 1:**
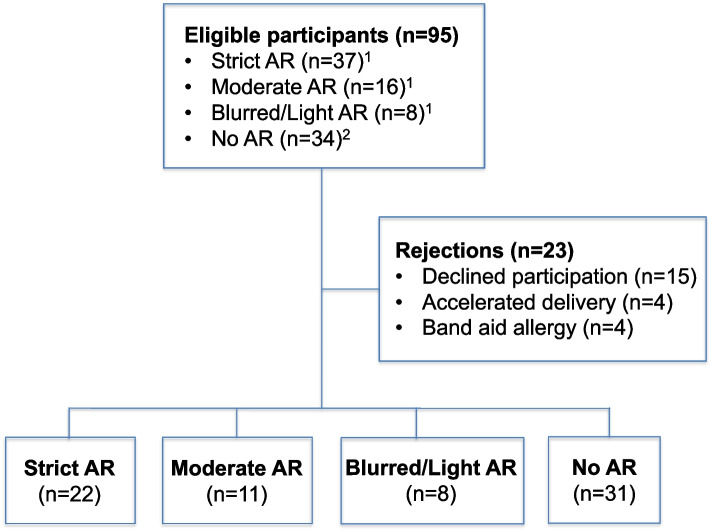
Flow chart of the study inclusion

In total 72 pregnant women were included; 41(57%) were recommended AR of whom 22(54%) had strict restrictions, 11(27%) moderate, 2(5%) light, and 6(14%) unspecified restrictions, whereas 31 participants (43%) had no restrictions (Table 1). A detailed description of inclusion flow is depicted in Fig. 1. The recommended ARs were indicated by one or more complications of preterm contractions, short cervix/threatened preterm delivery, vaginal bleeding, PPROM and/or other obstetric issues (Table 1). Of those recommended AR two participants had a cervical cerclage, whereas 5 had been treated with tocolysis prior to inclusion and 19 were treated at inclusion with vaginal progesterone (data not shown). All demographic and obstetric characteristics of the study participants are presented in Table 1.

**Table 1:** Demographic and obstetric characteristics of study participants stratified by recommended level of activity restriction (*n* = 72)

	**Total**	**Strict AR**	**Moderate AR**	**Light AR**	**Unspecified AR**	**No AR**
**Overall participants**, n (%)	72 (100)	22 (30.6)	11 (15.3)	2 (2.8)	6 (8.3)	31 (43.1)
**Maternal age (years)**						
median (min–max)	30.5 (22–43)	31 (22–43)	31 (23–42)	27.5 (25–30)	29.5 (26–31)	31 (23–41)
**Parity**						
Nulliparous, n (%)	39 (54.2)	11 (50)	6 (54.5)	1 (50)	4 (66.7)	17 (54.8)
**Gestational age at inclusion** (full weeks)						
median (min–max)	28 (22–33)	27 (22–33)	29 (22–32)	25 (25–25)	30 (24–33)	28 (22–32)
**Threatened preterm delivery complications**						
Preterm contractions, n (%)	5 (6.9)	4 (18.2)	1 (9.1)	0	0	0
Short cervix^a^ / threatened pretem delivery	26 (36.1)	13 (59.1)	9 (81.8)	1 (50)	3 (50)	0
Vaginal bleeding	4 (5.6)	2 (9.1)	1 (9.1)	0	1 (16.7)	0
PPROM	2 (2.8)	2 (9.1)	0	0	0	0
Other complications	5 (6.9)	1 (4.5)	0	1 (50)	2 (33.3)	1 (3.2)
None	30 (41.7)	0	0	0	0	30 (96.8)
**Pregestational Body Mass Index (BMI)** (kg/m2)						
median (min–max)	21.63 (17.84–49.67)	21.08 (17.84–49.67)	23.41 (18.51–35.76)	22.93 (19.61–26.26)	20.28 (19.27–21.10)	22.68 (18.61–36.83)
**Educational level** (years)						
≤ 12, n (%)	12 (16.7)	7 (31.8)	1 (9.1)	0	2 (33.3)	2 (6.5)
13–14	17 (23.6)	8 (36.4)	4 (36.4)	0	0	5 (16.1)
15 +	43 (59.7)	7 (31.8)	6 (54.5)	2 (100)	4 (66.7)	24 (77.4)
**Work status**						
Working, n (%)	28 (38.9)	2 (9.1)	1 (9.1)	0	1 (16.7)	24 (77.4)
Sick leave	38 (52.8)	20 (90.9)	9 (81.8)	2 (100)	3 (50)	4 (12.9)
Unemployed / Outside workforce	5 (6.9)	0	1 (9.1)	0	2 (33.3)	3 (9.7)
**Smoking status**						
Non-smoker, n (%)	68 (94.4)	20 (90.9)	11 (100)	2 (100)	5 (83.3)	30 (96.8)
**Partner status**						
Cohabitant with partner, n (%)	68 (94.4)	20 (90.9)	9 (81.8)	2 (100)	6 (100)	31 (100)
**Level of pregestional physical activity**^b^						
High, n (%)	3 (4.2)	1 (4.5)	0	0	0	2 (6.5)
Moderate	34 (47.2)	11 (50)	3 (27.3)	2 (100)	3 (50)	15 (48.4)
Light	33 (45.8)	8 (36.4)	8 (72.7)	0	3 (50)	14 (45.2)
Sedentary	2 (2.8)	2 (9.1)	0	0	0	0

*AR* Activity restriction

^a^Short cervix < 20 mm

^b^Self reported information

Among participants recommended strict, moderate, light or unspecified AR (*n* = 41) during data collection 16(39%) were inpatients, 13(32%) outpatients and 12(29%) were combined in-/outpatients (Table 2).

**Table 2:** Physical position and activity outcome stratified by recommended level of activity restriction

	**Strict AR**	**Moderate AR**	**Light AR**	**Unspecified AR**	**No AR**
**Overall**, n (%)	22 (30.6)	11 (15.3)	2 (2.8)	6 (8.3)	31 (43.1)
**Overall monitoring time**					
Monitored days, median (min–max)	6.96 (1.13–7.08)	6.96 (1.42–7.08)	7.04 (7.00–7.08)	6.98 (6.67–7.00)	7.04 (1.75–7.29)
**Physical positioning/activity** median hours pr day (min–max)					
Supine or lateral rest	17.70 (9.60–24)	15.13 (11.53–21.55)	14.23 (12.81–15.65)	14.73 (12.67–16.79)	10.54 (6.29–15.38)
Sitting upright	4.92 (0.11–11.69)	5.60 (1.99–9.29)	5.18 (5.05–5.30)	7.14 (3.99–8.10)	7.56 (0.05–11.36)
Standing upright	0.44 (0.06–1.13)	1.26 (0.27–2.39)	2.04 (1.28–2.79)	0.93 (0.34–1.61)	2.17 (0.98–3.89)
Sporadic gait	0.46 (0.09–1.05)	0.83 (0.22–1.86)	1.34 (0.96–1.73)	0.86 (0.33–1.67)	1.71 (0.80–3.02)
Walking	0.31 (0.01–1.17)	0.63 (0.11–1.49)	1.35 (1.21–1.50)	0.88 (0.15–1.83)	1.72 (0.65–3.42)
**Step count** median pr day (min–max)	1,520 (20–5,482)	3,310 (467–6,968)	7,544 (6,272–8,815)	4,751 (722–9,548)	9,235 (3,225–20,818)
**Admission status**, n (%)					
Inpatient	14 (63.6)	1 (9.1)	0	1 (16.7)	0
Outpatient	2 (9.1)	7 (63.6)	2 (100)	2 (33.3)	31 (100)
Combined in-/outpatient	6 (27.3)	3 (27.3)	0	3 (50.0)	0

*AR* Activity restriction

### Accelerometric results

**Fig. 2:**
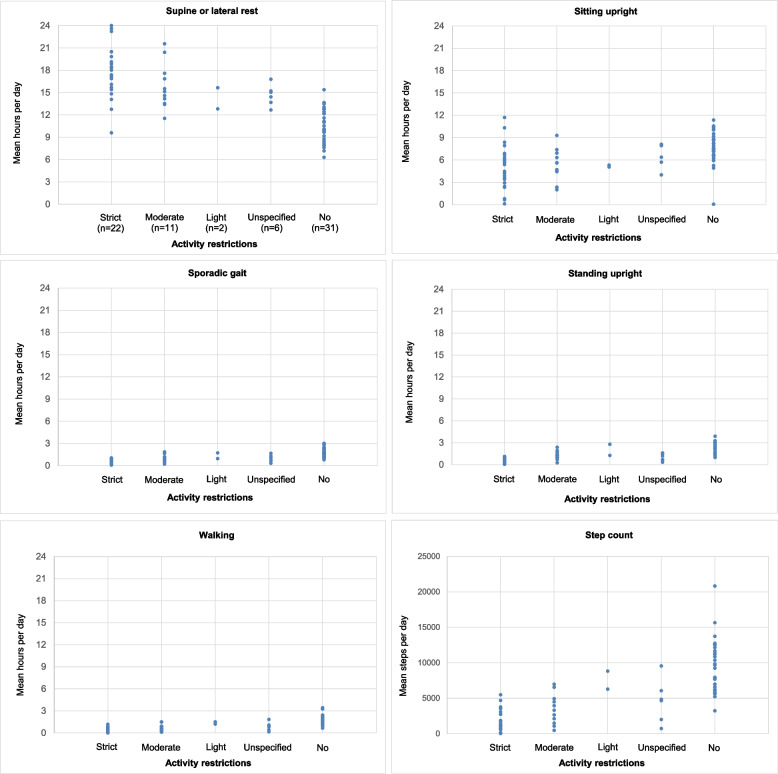
Individual performance of different daily physical positions/activities according to level of recommended activity restriction

Overall, participants with recommended strict/moderate AR spent more time in physically inactive, resting positions (supine or lateral rest and upright sitting) compared to no AR. Moreover, the intragroup variations of time spent in the inactive, resting positions, when standing upright, when walking and number of steps taken were high both within the different levels of prescribed AR as well as within the group of no AR (Fig. 2).

Women with no AR were all outpatients and spent 10.54 median hours/day (range: min.6.29-max.15.38) in the supine or lateral resting position; 7.56 h/day (0.05–11.36) sitting upright; stood upright 2.17 h/day (0.98–3.89) and took 9,235 steps/day (3,225–20,818) (Table 2).

In contrast participants with prescribed strict AR rested in the supine or lateral position for 17.7 h/day (9.60–24.0); sat upright 4.92 h/day (0.11–11.69); stood upright 0.44 h/day (0.06–1.13) and took 1,520 steps/day (20–5,482). Among the strict AR 63.6% were inpatients and took 1,155 steps/day (20–5,482) whereas 27.3% were combined in-/outpatients and took 1,803 steps/day (755–3,530) (Table 2).

Participants with moderate AR rested in the supine or lateral position for 15.13 h/day (11.53–21.55); sat upright 5.60 h/day (1.99–9.29); stood upright 1.26 h/day (0.27–2.39) and took 3,310 steps/day (467–6,968). Of those with moderate AR 63.6% were outpatients and took 3,944 steps/day (1,069–6,968), whereas 27.3% were combined in-/outpatients and took 2,112 steps/day (1,489–4,925) (Table 2).

Compared to participating women with no AR, women with strict/moderate AR were significantly more physical inactive both regarding time spent in supine or lateral rest (strict AR 6.58/moderate AR 4.75 more mean hours per day, both *p* < 0.001), sitting upright (3.51 (more)/-2.53 (less) mean hours per day, *p* = 0.006/ *p* = 0.011), standing upright (-1.28/-0.77 (less) mean hours per day, *p* < 0.001/ *p* = 0.003), sporadic gait (-0.84/-0.62 (less) mean hours per day, *p* < 0.001/ *p* = 0.014) and walking (-0.89/-0.79 (less) mean hours per day, *p* 0.001/ *p* < 0.001). Moreover, the women with strict/moderate AR took -5,040/-4,592 (less) mean steps (*p* 0.001/ < 0.001) compared to those with no restrictions (Table 3). These findings are visually illustrated at individual participant level in Fig. 2.

**Table 3:** Linear regression on associations between physical positions/activities and recommended AR (strict or moderate AR versus no AR)

	Supine or lateral rest^a^ β [95% CI]	***p***	Sitting upright^a^ β [95% CI]	***p***	Standing upright^a^ β [95% CI]	***p***	Sporadic gait^a^ β [95% CI]	***p***	Walking^a^ β [95% CI]	***p***	Step count^c^ β [95% CI]	***p***
**Physical positioning/activity**												
**Unadjusted model**												
Strict AR, (*n* = 22)	7.13 [5.59; 8.68]	< 0.001	2.66 [-3.99; -1.33]	< 0.001	-1.68 [-2.03; 1.33]	< 0.001	-1.27 [-1.54; -1.00]	< 0.001	-1.39 [-1.69; -1.10]	< 0.001	-7,626 [-9,231; -6,021]	< 0.001
Moderate AR, (*n* = 11)	5.18 [3.24; 7.13]	< 0.001	-2.29 [-4.00; -0.61]	0.008	-0.95 [-1.39; -0.51]	< 0.001	-0.80 [-1.14; -0.46]	< 0.001	-1.06 [-1.42; -0.69]	< 0.001	-5,979 [-7,999; -3,958]	< 0.001
**Adjusted model**^b^												
Strict AR, (*n* = 22)	6.58 [3.78; 9.39]	< 0.001	3.51 [-5.95; -1.07]	0.006	-1.28 [-1.93; -0.63]	< 0.001	-0.84 [-1.33; -0.35]	0.001	-0.89 [-1.41; -0.37]	0.001	-5,040 [-7,929; -2,151]	0.001
Moderate AR, (*n* = 11)	4.75 [2.55; 6.96]	< 0.001	-2.53 [-4.44; -0.61]	0.011	-0.77 [-1.28; -0.26]	0.003	-0.62 [-1.01; -0.23]	0.014	-0.79 [-1.19; -0.02]	< 0.001	-4,592 [-6,862; -2,322]	< 0.001
**No AR, (*n* = 31)**	reference		reference		reference		reference		reference		reference	

*AR* Activity restriction, *CI* Confidence interval

^A^mean hours per day

^B^linear regression mutually adjusted for admission status, gestational age, parity, monitoring time and educational status

^C^mean steps per day

No differences were found between the two groups (strict vs. moderate AR) in the time spent resting in the supine/lateral position (*p* = 0.199), when sitting upright (*p* = 0.492) nor in the number of steps taken (*p* = 0.188). However, the women stood less time upright (*p* = 0.016) when recommended strict AR (Table 4).

**Table 4:** Linear regression on associations between physical positions/activities and recommended AR (strict versus moderate AR)

	Supine or lateral rest^a^ β [95% CI]	***p***	Sitting upright^a^ β [95% CI]	***p***	Standing upright^a^ β [95% CI]	***p***	Sporadic gait^a^ β [95% CI]	***p***	Walking^a^ β [95% CI]	***p***	Step count^b^ β [95% CI]	***p***
**Physical positioning/activity**												
**Unadjusted model**												
Strict AR, (*n* = 22)	1.95 [-0.58; 4.48]	0.126	-0.38 [-2.45; 1.70]	0.714	-0.73 [-1.07; -0.39]	< 0.001	-0.46 [-0.73; -0.19]	0.001	-0.34 [-0.61; -0.07]	0.017	-1,647 [-2,958; -336]	0.015
**Adjusted model**^c^												
Strict AR, (*n* = 22)	2.12 [-1.19; 5.44]	0.199	-0.95 [-3.75; 1.85]	0.492	-0.59 [-1.07; -0.12]	0.016	-0.33 [-0.68; 0.02]	0.067	-0.23 [-0.58; 0.13]	0.199	-1,119 [-2,823; 584]	0.188
**Moderate AR, (*n* = 11)**	reference		reference		reference		reference		reference		reference	

*AR* Activity restriction, *CI* Confidence interval

^a^mean hours per day

^b^mean steps per day

^c^linear regression mutually adjusted for admission status, gestational age, parity, monitoring time and educational status

**Fig. 3:**
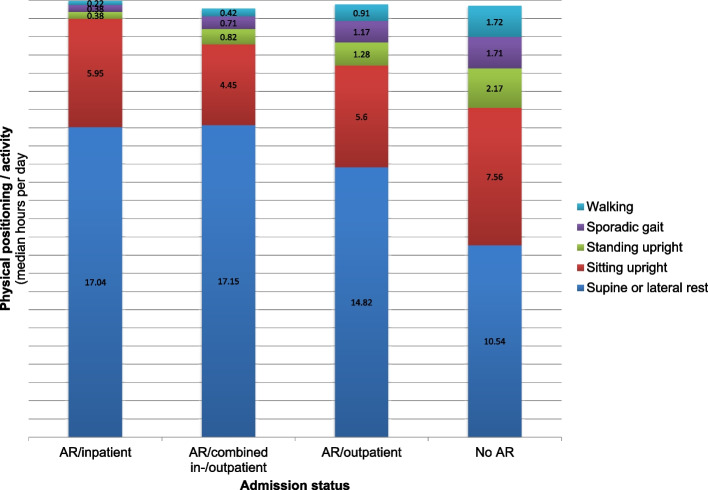
Physical positions/activities according to admission status

The duration of time spent in the different physical positions and activities according to admission status is illustrated in Fig. 3. Moreover, when recommended strict AR no significant differences were found between admission status in the time spent in any of the different physical positions and activities, nor in the number of steps taken (Table 5).

**Table 5:** Linear regression on associations between physical positions/activities and admission status when recommended strict AR

	**Supine or lateral rest**^a^ β [95% CI]	p*	**Sitting upright**^a^ β [95% CI]	p*	**Standing upright**^a^ β [95% CI]	p*	**Sporadic gait**^a^ β [95% CI]	p*	**Walking**^a^ β [95% CI]	p*	**Step count**^b^ β [95% CI]	p*
**Combined i/o** (*n* = 6)	1.21 (-2.50; 4.91)	0.216	-1.67 (-4.72; 1.38)	0.138	0.12 (-0.27; 0.50)	0.364	0.17 (-0.13; 0.46)	0.284	0.07 (-0.26; 0.40)	0.409	301 (-1,356; 1,958)	0.322
**Inpatient** (*n* = 14)	reference		reference		reference		reference		reference		reference	

*AR* Activity restriction, *i/o* Inpatient/outpatient, *CI* Confidence interval

^*^Mann–Whitney U test

^a^mean hours per day

^b^mean steps per day

## Discussion

We found as expected that participating women recommended strict and moderate AR spent more time in the physical inactive, resting positions than pregnant women with no restrictions. However, women within the same level of AR performed the restrictions with extensive variability. Moreover, only the time standing upright differed between strict and moderate AR and the admission status did not affect adherence to the recommended level of AR.

In our study, inpatients recommended strict AR took 1,155 median steps per day, which was higher than demonstrated in an American pilot intervention study, where 11 participants who were recommended bed rest took only 200 median steps per day, and 11 without restrictions took 837 steps [23]. The American pilot study included 35 hospitalized women with PPROM at gestational age less than 34 full weeks that were randomized to either strict bed rest (verbally instructed to spent majority of their day in the hospital bed) or to an unrestricted group (verbally instructed to move without limitations and as a minimum to walk around 20 min three times a day) [23]. In contrast, we included only two participants with PPROM, whereas the main indication for strict AR was short cervix/threatened preterm delivery. If recommended strict AR the participants in our study had been instructed to rest in bed or in a sitting position most of the day (> 8 h of the awaken hours of the day) except for meals, bathing, and bathroom use. The differences in step counts between these studies may possibly be a consequence of both variations of the pregnancy complications, in the AR patient instructions as well as in general patient adherence and culture.

Our study demonstrated no significant differences in any of the physical positions / activities or step counts between the strict and moderate activity restricted groups except time spent in the standing upright position, where the strict group stood upright 0.73 less median hours per day than the moderate group. This may question if it makes clinical sense to differentiate between strict and moderate AR. Moreover, participants who were recommended the same level of AR did adhere to the prescription with extensive variations, especially when resting in the supine or lateral position. It may indicate that an accurate dosage of physical positioning and activity is not definite and may be influenced by the clinician’s instructions as well as participants’ individual interpretation. On the other hand, we demonstrated as well a great variation in how uncomplicated pregnant women with no physical AR moved around in their daily lives. This may verify that pregnant women in general perform their daily physical positions and activities with great variations regardless of being recommended AR or not.

Being recommended AR is a significant personal intervention [24–26] hence the adherence to the AR recommendation may be powered by both individual motivators and barriers [27]. It is therefore important that recommended AR is explicitly specified yet individually attainable. In our study 15% of participants perceived being prescribed unspecified AR. This indicates the importance of more precise and unambiguous AR patient instructions, which also seems to be emphasized in the literature by an identified lack of professional consensus how to define and label different levels of AR [1, 12].

Moreover, it is essential that healthcare professionals acknowledge that several challenging dimensions of being recommended AR are at stake. In order to reach an intrinsic, meaningful and self-determined experience of the AR and thereby being able to adhere to the recommendation the pregnant woman must be guided and supported individually [27].

There is a lack of data definitively demonstrating that AR improves perinatal outcomes in pregnancies complicated not only by threatened preterm delivery but also in multiple pregnancies, fetal growth restriction, PPROM, or hypertensive diseases of pregnancy [14, 28, 29]. Hence the use and the effects of AR has repeatedly been debated [1, 10, 30]. Lately, the Society for Maternal–Fetal Medicine has unequivocally recommended against the routine use of any level of physical AR as a means to prevent preterm delivery in case of preterm labor symptoms, arrested preterm delivery, and shortened cervix [9]. Rather several studies have shown a preventive tendency of physical activity on risk of preterm delivery primarily in cases of short cervix [30, 31]. Thus, in clinical practice a caution should be that AR is not recommended routinely but only in rare cases of immediate risk or complications known to be treatable with physical inactivity. However, from our clinical experiences there is still an ongoing tendency to recommend AR despite the general counsel against routine AR recommendations [8, 9]. Presumably, if clinicians are to discontinue the routine practice of AR in threatened preterm deliveries a randomized controlled study is still needed to evaluate the treatment effects of AR. The results from our study are extremely valuable in such future research studies when designing realistic interventions of AR.

Our study demonstrated that even healthy pregnant women with no AR in some cases spent many more hours in physical inactive positions than were to be expected. It has repeatedly been demonstrated that physical activity in healthy pregnant women reduces the risk of pregnancy related complications such as preterm delivery, preeclampsia, hypertensive disorders, gestational diabetes, and fetal growth restriction [8, 32–35]. This calls for an important general attention in the antenatal care to encourage a physical active lifestyle during all stages of healthy pregnancies, as an early prevention strategy is preferable and may reduce the risk of pregnancy related complications.

Our study contributes with new knowledge about how much time is daily spent in physical resting positions and activities both by women with recommended AR during pregnancy and by women without any restrictions, using chest and knee worn tri-axial accelerometry. This study is to our knowledge the first of its kind in the obstetric field. Other studies have used knee worn tri-axial accelerometry performed in other health care fields to detect changes in physical inactivity during training sessions as well as during different kinds of hospitalizations [36, 37].

Our accelerometric data were validated based on a qualitative audit process with excellent agreement in all tested cases, which was a strength. A limitation was however, the rather small study population. When the accelerometric data were stratified in the different AR groups, the relative low number of observations included in the adjusted linear regression models may have involved some dilution of the data information.

Moreover, it is a limitation of this study that the data collection did not discriminate between sleep and awake, supine or laid-back rest and also that the SENS monitors used to collect the accelerometric data have never been validated on a pregnant population.

## Conclusion

Overall, participants adhered highly to the recommended AR. However, discriminating between strict and moderate AR recommendations did not alter how physical resting positions and activities were carried out. The admission status did not influence how participants adhered to strict AR.

## Data Availability

The datasets generated and/or analysed during the current study are not publicly available due to confidentiality but are available from the corresponding author on reasonable request. We can transfer individual participant data only when we have obtained approval from the Danish Data Protection Authority according to the Data Protection Act and completed a Standard Contractual Clause to ensure the legal basis of the transfer.

## Abbreviations

AR: Activity restrictions
PPROM: Preterm prelabor rupture of membranes
